# Community engagement initiatives in primary health care to achieve universal health coverage: A realist synthesis of scoping review

**DOI:** 10.1371/journal.pone.0285222

**Published:** 2023-05-03

**Authors:** Daniel Erku, Resham Khatri, Aklilu Endalamaw, Eskinder Wolka, Frehiwot Nigatu, Anteneh Zewdie, Yibeltal Assefa

**Affiliations:** 1 Centre for Applied Health Economics, School of Medicine, Griffith University, SouthPort, Queensland, Australia; 2 Menzies Health Institute Queensland, Griffith University, SouthPort, Queensland, Australia; 3 School of Public Health, The University of Queensland, Brisbane, Queensland, Australia; 4 College of Medicine and Health Sciences, Bahir Dar University, Bahir Dar, Ethiopia; 5 International Institute for Primary Health Care in Ethiopia, Addis Ababa, Ethiopia; Deakin University, AUSTRALIA

## Abstract

**Background:**

Community engagement (CE) is an essential component in a primary health care (PHC) and there have been growing calls for service providers to seek greater CE in the planning, design, delivery and evaluation of PHC services. This scoping review aimed to explore the underlying attributes, contexts and mechanisms in which community engagement initiatives contribute to improved PHC service delivery and the realisation of UHC.

**Methods:**

PubMed, PsycINFO, CINAHL, Cochrane Library, EMBASE and Google Scholar were searched from the inception of each database until May 2022 for studies that described the structure, process, and outcomes of CE interventions implemented in PHC settings. We included qualitative and quantitative studies, process evaluations and systematic or scoping reviews. Data were extracted using a predefined extraction sheet, and the quality of reporting of included studies was assessed using the Mixed Methods Appraisal Tool. The Donabedian’s model for quality of healthcare was used to categorise attributes of CE into “structure”, “process” and “outcome”.

**Results:**

Themes related to the structural aspects of CE initiatives included the methodological approaches (i.e., format and composition), levels of CE (i.e., extent, time, and timing of engagement) and the support processes and strategies (i.e., skills and capacity) that are put in place to enable both communities and service providers to undertake successful CE. Process aspects of CE initiatives discussed in the literature included: i) the role of the community in defining priorities and setting objectives for CE, ii) types and dynamics of the broad range of engagement approaches and activities, and iii) presence of an on-going communication and two-way information sharing. Key CE components and contextual factors that affected the impact of CE initiatives included the wider socio-economic context, power dynamics and representation of communities and their voices, and cultural and organisational issues.

**Conclusions:**

Our review highlighted the potential role of CE initiatives in improving decision making process and improving overall health outcomes, and identified several organisational, cultural, political, and contextual factors that affect the success of CE initiatives in PHC settings. Awareness of and responding to the contextual factors will increase the chances of successful CE initiatives.

## Background

Primary health care (PHC), which first came to the fore with the 1978 Alma-Ata declaration [1], provides the programmatic engine for universal health coverage (UHC) in most contexts and countries [2]. Since the Alma Ata declaration, community engagement (CE) has become a central tenet in a PHC, and there have been growing calls for service providers to seek greater CE in the planning, design, delivery and evaluation of PHC services [3]. CE is an essential feature in the World Health Organisation (WHO)’s Framework on Integrated, People-centred Health Services [4], and member states reaffirmed their commitment to empower individuals and communities at the signing of the Astana Declaration [5].

Communities often have a more holistic view of health and wellbeing; engaging communities proactively in the planning, design, delivery, and evaluation of PHC services can lead to improved community health [6]. Clear, structured plans guide successful community engagement initiatives with respect to the aim, content, and level of engagement, as well as approaches for addressing power imbalance, representation, and transparency [6–8]. One of the key aspects of CE is the use of appropriate levels of engagement. The extent or levels of CE can be considered a spectrum, progressing from least engagement (receiving information) to active engagement (control and empowerment). PHC service providers in many countries are implementing CE initiatives, albeit with varying levels of engagement [9–14]. Several frameworks exists to describe the extent (levels) of CE, the most common being the International Association for Public Participation’s public participation spectrum (*Inform*, *Consult*, *Involve*, *Collaborate*, *and Empower*), Arnstein’s Ladder of Citizen Participation (participation ranging from meaningless and tokenistic participation to empowerment) [15] and Draper *et al* three-level CE framework (*Mobilisation*, *Collaboration*, *and Empowerment*) [16, 17]. Other conceptualisations of CE are also widely utilised, including Travaglia and Robertson’s three levels of CE (micro; meso; and macro [18], and Bowen et al. [19]. “Continuum of community engagement” framework, in which engagement strategies are categorised into “transactional, transitional, and transformational engagement”. While these frameworks used varying terminologies to describe levels of CE, the fundamental logic underpinning these frameworks is the extent of involvement of communities and spectrum of control or influence on decision-making processes. The community’s level of involvement can be influenced by what it entails (i.e., definitions of community and community engagement) and wether it is aligned with and responsive to the needs and motivations of communities [20].

Despite the wide acceptance of CE in theory and practice and long history of CE research, there is little concrete evidence on the effectiveness of CE initiatives implemented in PHC settings, and there is no clear assessment of the contextual factors and mechanisms for effective CE in PHC setting [21–23]. Specifically, previous studies provided limited insight into the role of contextual factors and underlying mechanisms in influencing CE intervention outcomes [8, 9, 24–26]. By understanding why, how, for whom, and in what circumstances CE work (or do not), policy makers and program implementors can adapt and adopt successful CE initiatives into their health system contexts. Using Donabedian’s model for quality of healthcare [27, 28] and a realist synthesis approach, this scoping review examined CE initiatives’ key structural and process features that lead to better health outcomes in PHC settings. Realist review is a methodology that aims to understand the underlying mechanisms that drive the outcomes of complex interventions. It seeks to identify and explain how and why an intervention works (or doesn’t work), by examining the contextual factors and underlying mechanisms that influence its effectiveness or ineffectiveness [29, 30]. The process of realist review involves a systematic and iterative approach to identify and test theories about what works, for whom, and in what circumstances [30, 31]. This methodology is particularly valuable for evaluating complex interventions, such as community engagement initiatives, that involve multiple components, stakeholders, and contextual factors. By using realist review, researchers can identify the specific components or elements of the intervention that are effective in particular contexts or for specific populations.

## Methods

We conducted a scoping review of published evidence reporting community engagement initiatives in the context of PHC and UHC. The review was conducted following the Preferred Reporting Items for Systematic Reviews and Meta-Analyses extension for scoping reviews (PRISMA-ScR) guideline [32]. A comprehensive description of our methodology can be found in Erku et al [33].

### Data sources and search strategy

We searched six electronic databases (PubMed, CINAHL, Web of Science, Cochrane Library, EMBASE, and Google Scholar) and grey literature for studies that described the structure, process, and outcomes of CE interventions implemented in PHC settings. This was followed by complementary searches, including forward and backward citation searches of included studies, and Google searches to further locate eligible articles that were not identified in the database searches. The keywords used in the search strategy were built on three key concepts (community engagement, primary health care, and universal health coverage), and tailored to each database (see S1 Appendix). Boolean operators and truncations varied depending on the database. The search included articles published in English language from inception of each database up to the 29^th^ of May 2022 (and again in April 2023). No time- or country-related limitations were applied.

### Eligibility screening

We included qualitative and quantitative studies, programme manuals and systematic reviews, editorials, opinion/ position pieces and process evaluations that reported data on structure, process and outcomes of CE initiatives, including contextual factors affecting the acceptability, feasibility, and implementation of CE. In this paper, community engagement is defined as a process whereby PHC service system: i) proactively seeks out and incorporate community values, needs and motivations into a decision-making process, and ii) establishes an ongoing partnership with the community to ensure that service delivery is aligned with, and continue to be shaped by community’s values and needs. We have not specified CE to a particular level of engagement. We excluded community engagement interventions specific to a health condition. We also excluded conference or dissertation abstracts without the full text available for retrieval. The articles identified were then exported to COVIDENCE (Veritas Health Innovation Ltd), and two independent reviewers screened all titles, abstracts and full texts based on the eligibility criteria. Any discrepancies or disagreements between reviewers were resolved through discussion to reach a consensus. Quality appraisal took an iterative, holistic approach consistent with the realist synthesis approach and was conducted throughout the review process [29]. The purpose of the quality appraisal was to interpret the findings in light of the quality of the included studies, rather than as inclusion criteria.

### Data extraction and synthesis

Evidence from each document was abstracted and synthesised using deductive and inductive approaches. The data extraction process was iterative, with repeated discussion (and consensus where there are disagreements) among the research team on data extraction approach and the initial analytical framework. We extracted information related to study details (e.g., authors, year of publication, study aim and design, and participant characteristics) and key findings (e.g., aims, type, and area of community engagement intervention, and impact of CE including mechanisms describing how CE influenced PHC attributes and outcomes related to UHC). We employed Donabedian’s model for quality of healthcare [27, 28] to categorise attributes of CE in PHC into the three dimensions: “structure”, “process” and “outcome”. Such attribute classification system provides an opportunity to group a wide range of CE features. Attributes related to “structure” refer to the settings or contexts within which CE occurs, and include any political, legal, professional or personal resources and organisational structure. “Process” entails all activities pertaining to the methods or mechanisms by which CE occurs, and the “outcomes” dimension includes attributes related to the effect of engagement activities. Community engagement initiatives may work in one context but not in others, and it is important to understand the context and mechanisms in which such initiatives are implemented. Thus, we followed a realist evaluation approach to unpack the heterogeneity and complexity of CE interventions, thereby understanding what works for who and under what circumstances. Where studies conducted in comparable circumstances or contexts reported differing findings, the sources of evidence were consolidated and situated to explain possible reasons. We also juxtaposed sources of evidence in situations where information about community engagement initiatives in one document allows insights into evidence about outcomes in another document.

## Results

**Fig 1:**
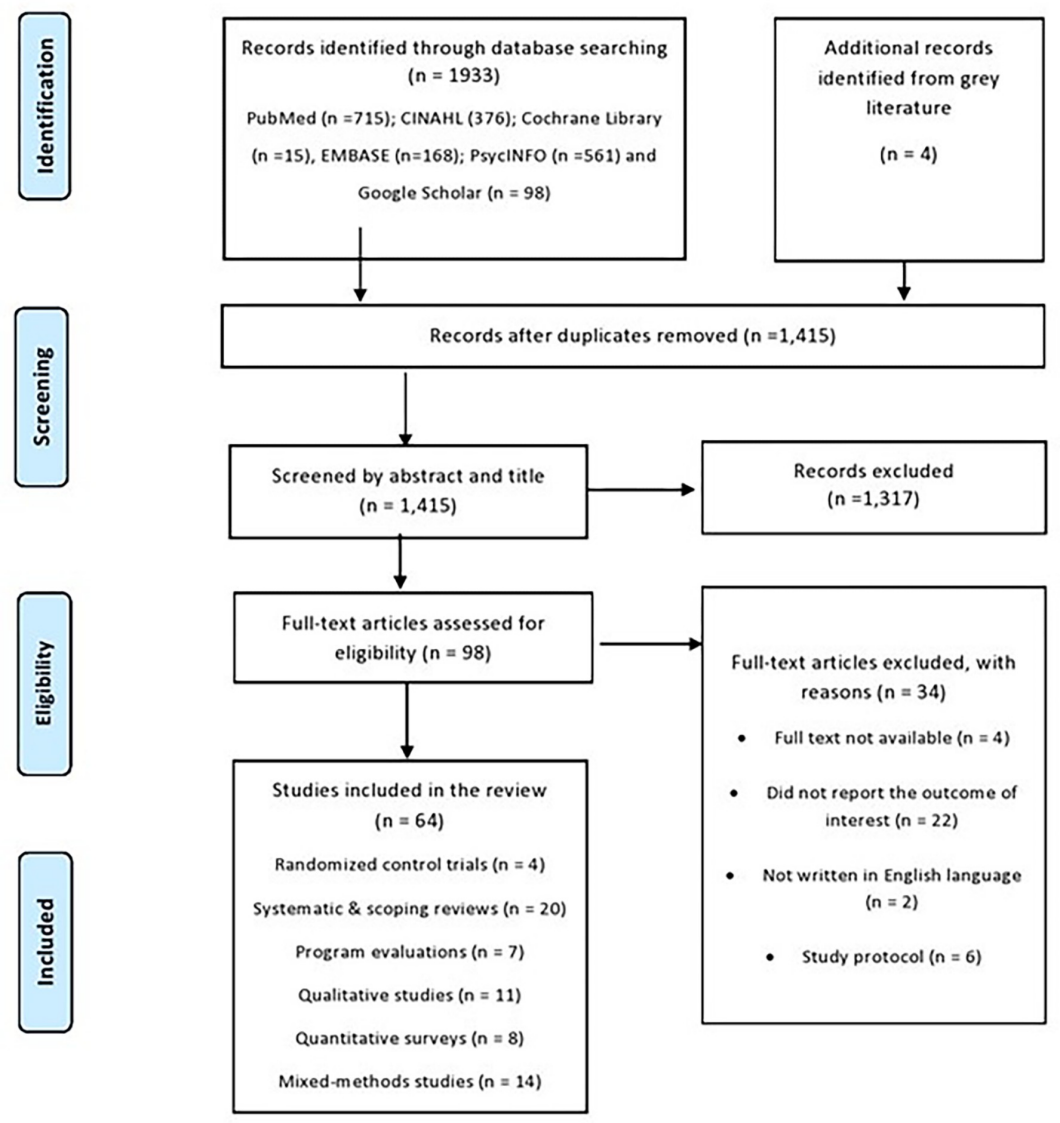
Preferred Reporting Items for Systematic Reviews and Meta-Analyses (PRISMA) flow diagram.

After removal of duplicates and publications that did not meet the inclusion criteria, we included a total of 67 studies conducted in 21 countries including systematic and scoping reviews [13, 16, 21, 23–26, 34–46], randomised control trials [47–50], program evaluations and case studies [7, 51–56], qualitative studies [6, 10, 11, 20, 57–63], quantitative surveys [9, 64–70], and mixed-methods studies [8, 12, 14, 71–79]. The detailed search strategy and eligibility screening are presented in Fig 1, and detailed description of each article is presented in S1 Appendix.

### Structural aspects of CE initiatives in PHC

#### The level, format, and composition of engagement

Many studies in our review described different levels of community engagement, from passive and tokenistic participation to more active involvement on community empowerment. The impact of CE initiative in achieving stated objectives (e.g., improving service uptake, health outcome) was partially contingent on whether communities were engaged at the appropriate level of participation with adequate engagement time, duration, and frequency as well as appropriate timing [21, 39, 80]. In a national study conducted in New Zealand to examine the process of involving communities in PHC, various stakeholders clearly distinguished community participation from consultation. The latter was described as formal participation with little or no influence on ultimate decision making [6]. CE initiatives target a broad range of individuals and groups, ranging from those who represent themselves, and specific communities (e.g., indigenous groups, and culturally and linguistically diverse communities) to those who are asked to represent consumers [21–23, 36, 51, 56]. CE initiatives for these community groups involved different aims, challenges, and strategies. For example, indigenous groups’ use of indigenous health workers were identified as essential for an effective engagement process [56]. Gender and ethnic diversity as well as inclusion of people from lower socio-economic backgrounds had implications for participation in CE activities [21–23, 36, 51]. None of the studies in this review explored diversity in representation (e.g., the extent to which women are adequately represented and actively participated in decision-making within decision-making groups). Rather, studies reported the gender composition within decision-making groups [16], and all studies that mentioned gender composition reported that men dominated membership and leadership within decision-making groups [16, 55, 64]. A review by Karuga *et al* reported that the selection processes of community-level health committees in Sub-Saharan African countries were not transparent and participatory, resulting in a lack of legitimacy [16].

#### Skills and capacity to conduct CE

This theme involves support processes and strategies that are put in place by relevant authorities to enable both communities and staff/professionals to undertake successful CE [40]. This includes but not limited to availability of resources (funding, incentives) and facilitative leadership, training and capacity building, information technology infrastructure, and an engagement-capable environment (i.e., physical or virtual location and socio-cultural conditions such as reciprocity, culturally sensitive and open communication). All these factors contribute not only to the short-term success of CE in PHC but also long-term effectiveness and sustainability of such initiatives. Several studies reported that short-term success, long-term effectiveness, and sustainability of CE initiatives depends largely on adequate training of all stakeholders involved in delivering CE, including communities, staff, and service providers [40, 76]. In addition, an engagement-capable environment that supports communities and removes engagement barriers–particularly for marginalised people and those with disability–is considered essential for the success of CE activities about the issues of equity in access to PHC services [80]. In many sub-Saharan African countries, community groups such as health committees played a crucial role in mobilising resources to support PHC delivery through various means including donations, organising community members to contribute in-kind (time, skills, raw materials etc), and lobbying local managers to retain health workers and support staff in their PHC facilities [16]. Overall, studies consistently reiterated the importance of i) collective and participatory decision-making, ii) adequate participant and provider skills training and administrative support, iii) adequate engagement time, duration, and frequency as well as appropriate timing, and iv) cash flow stability throughout the lifetime of a CE initiative.

### Process aspects of CE initiatives in PHC

Several examples of process issues were discussed in the literature, including: i) clearly defined community-informed objectives, ii) types and dynamics of the broad range of engagement approaches and activities, and iii) presence of an ongoing communication and two-way information sharing.

#### The role of the community in defining priorities and setting objectives for CE

The extent to which the community is allowed to define priorities and set the objectives and agenda of engagement is one of the essential process aspects of CE initiatives. The literature reviewed consistently highlighted the importance of stakeholder collaboration–based on the shared values and vision–in developing the objectives and agenda for CE activities [21, 25, 39, 80, 81]. Among the most cited objectives for CE includes improving the level of general and specific PHC delivery, addressing healthcare access issues, particularly for disadvantaged people, and prevention and screening campaigns (e.g., HIV/AIDS, breast cancer screening) [21, 24, 25, 36, 38, 44]. The need for a strategic policy direction to embed community participation as a core principle underpinning the development of PHC was one of the main lessons learnt in Ireland’s Joint Community Participation in Primary Care Initiative [82]. Peru’s Local Health Administration Communities (CLAS) is another example of this approach, with non-profit associations partnering with the government to oversee PHC service delivery using public funds [83, 84]. Through the implementation of CLAS, administrative power for rural health services was transferred from state governments to local communities, enabling social control over finance and health personnel. They manage their resources in a private bank account and can hire health workers based on performance, allowing for flexible financial management with social participation. Increased flexibility also leads to locally appropriate incentives that increase accountability, decentralize personnel control, and meet community demands [84]. As a result, CLAS improves PHC accessibility, resulting in increased utilization, coverage of essential services, and better outcomes [83]. Another example is USA’s Federally Qualified Health Centers (FQHCs), a community-based primary care clinics that provide comprehensive primary care services to underserved populations, including low-income families, rural communities, and individuals without health insurance. Despite being required to have a governing board with at least 51%consumers, a study by Wright have shown that a minority of board members are representative consumers, leading to significant socioeconomic gaps between the board members and FQHC patients [85]. This lack of representation can result in FQHCs being less responsive to the needs of low-income communities [85].

#### Engagement approaches and activities

The types and dynamics of engagement activities reported included planning, designing, governing, and evaluating PHC services; developing guidelines; allocating resources; reconfiguring health services; setting priorities; and providing feedback on documents or processes. We explored the extent to which community representatives (e.g., health committees) provided leadership in decision making across these CE steps and found that while they were involved in voicing their communities’ concerns about the quality PHC care provided and in making decisions related to day-to-day management of PHC facilities, they were left out in planning and budgeting processes [16]. Our review did not find studies documenting community group involvement in participatory evaluation of PHC services that produce meaningful local feedback. Rather, community representatives often monitored the quality of PHC provided by health workers, drug stocks and financial records in the facility.

#### Building rapport and communication

Building relationships based on mutual trust and meaningful exchange of ideas and information were reported to be of particular importance for marginalised groups [12, 54, 56, 86]. Studies also highlighted that the encounter between PHC providers and community requires cultural competence–the ability of providers to translate information in a way that gives due consideration to health literacy level and encourages community understanding [44, 54]. Several reviews included in our study reiterated that for CE activities to be meaningful and lead to positive change for communities, they need to be based on transparency, trust-building and information-sharing between communities and service providers, as this will enable people to feel comfortable seeking PHC services while providing PHCs with the opportunity to align services to community’s need. Changes in communication media and methods have increased the opportunity for the engagement of communities across PHC delivery modes. Decision aid tools such as information sheets, leaflets and videos were used to provide structured information about health options and support participation and decision-making process [65].

### Outcomes of CE initiatives in PHC setting

#### The impact of CE initiatives in improving decision-making process

CE provides the opportunity for communities to have a substantive influence on decision-making process. Most studies reported more than one outcome measure on the quality of PHC, including enhanced service delivery [66], and development of specific policy or planning documents [20, 39, 68, 76]. Several systematic reviews demonstrated that a participatory decision-making process that is built on mutual trust and understanding (i.e., one that gives due consideration to the needs, insights, and firsthand experiences of communities) have resulted in shaping policies, service deliveries, priorities, processes, guidelines, and other PHC related initiatives [21–24, 37, 40, 44, 81]. CE initiatives such as health facility committees were also found to be effective in facilitating social accountability by engaging with health providers in person or through meetings to discuss service failures, leading to changes in the quality of services [58]. CE initiative also led to organisational redesign and the delineation of roles and responsibilities between Aboriginal community-controlled health service and local Australian health service, and resulted in significant structural changes to how, where and who delivered PHC service among Australian Aboriginal communities [12]

#### The impact of CE initiatives in improving health outcome

Improved accountability and decision-making processes, realised through CE, will improve the quality of PHC delivered. Several studies consistently demonstrated the impact of CE initiatives in improving health outcomes [12, 34, 36, 47–49, 52, 64]. The evidence around the impact of CE on intermediate outcomes such as access to services, utilisation and quality is also stronger in the Aboriginal community-controlled sector [12]. In a study conducted in three African countries to examine the impact of community-directed intervention (CDI) on service coverage, it was reported that CDI approaches resulted in significantly higher coverage of PHC services at a low cost [26]. Central to this outcome was the participatory nature of the process and commitment of communities and community implementers [52]. Interestingly, community implementers were more motivated by intangible incentives than external financial incentives. This finding was reported in other studies [26]. Interestingly, no studies reported on outcomes relating to cost-effectiveness of real-world CE initiatives in PHC, despite the importance of such evidence in identifying CE initiatives that can achieve the greatest health return on investment [24]. Furthermore, sustainability and succession planning seemed absent across and within included studies.

### Contextual factors and mechanisms leading to successful CE in PHC

Convergent evidence from included studies suggests a common set of characteristics that underpin effective CE [21–25, 36–38, 40, 44, 81]. Key CE components that affected health outcomes included the wider socio-economic context, power dynamics and representation of communities and their voices, and cultural and organisational issues [12, 21–25, 36, 37, 40, 51]. A study by Scott et al investigated the contextual factors that affected the effectiveness of Village Health, Sanitation and Nutrition Committees in rural north India [87]. While technical inputs to improve committee form and functioning were successful, participant accounts revealed how social hierarchies, power dynamics, and resource and capacity deficits limited the committees’ efficacy. Fragmented administrative accountability and narrow authority further hampered committee members’ ability to involve diverse government services across health, sanitation, and nutrition sectors [87]. Several studies reported the impact of the wider political environment in facilitating or hindering CE. Socio-political contexts can directly influence the degree of social cohesion, trust and collective identity within a given community, with a direct implication on the success of CE initiatives. The inter-linked challenges of political commitment and resources are important factors to consider since CE can only be sustainable when relevant stakeholders remain committed, and there is a conducive socio-political and economic environment. For example, policy decisions regarding resource allocation within the wider health system can also directly impact the funds available for conducting effective CE activities within PHC settings. In one study conducted in Ireland, CE participants felt it was legitimate to be involved in the process because CE formed part of the national primary healthcare strategy [20]. Colombia’s health insurance system includes a legal and regulatory provision that allows citizens to form health insurance user associations to represent their interests to health insurance companies. However, despite having a mandate to represent citizens’ interests, enable participation in insurer decision-making, defend users, and oversee quality services, many user associations throughout Colombia are weak, passive, or inactive, and their existence is widely unknown to the public. A recent study identified low public awareness as a contextual factor that has impacted the functionality of these associations, thereby limiting their ability to empower citizens and influence health insurance responsiveness [79].

Similarly, aligning a strong local community and health service vision with the government’s health policies and priorities was reported as one of the main enabling factors for a successful CE among Australian Aboriginal communities [12]. National and international partners and lobbyists can also contribute to the success of CE. In terms of implementing organisation, using pre-existing organisations already put in place within the community (e.g., women’s groups and micro–credit savings groups [51, 82] may have more trust and legitimacy than a new service created for the purpose of community engagement. Similarly, pre-existing social structures and networks (e.g., those found in rural areas), local infrastructure and geographical accessibility were all important factors in success [82]. Importance of understanding CE as a political activity was also highlighted in a study conducted in Colombia in which the history and power of communities with different ethnic-rural territories were important elements in the formation of and involvement in community participation [72]. In South Africa, participation in CE is seen as a route to decolonisation, reflective of social justice paradigm and further underscoring the influence of history and culture in community participation [53].

Sustainability and functionality of CE initiatives were also reported as critical challenges for a long-lasting, meaningful participation [61] and are mainly impacted by lack of resources and poor organisational structure. Poor health literacy, which includes individual’s lack of capacity to navigate the health system’s organisational environment, greatly impacts people’s capacity to access, understand, appraise and apply health-related information [73]. Thus, capacity building and support for community members is crucial to assist community members to understand the system or service bureaucracy and to contribute meaningfully to CE. In addition to the aforementioned contexts and mechanisms, adequate mobilisation and advocacy for CE programs [66] and prior experience of successful community participation in PHC [20] determines effectiveness of CE initiatives. Aboriginal community-controlled health services in Australia [12, 86], Ireland’s Joint initiative on Community Participation in PHC [60, 82], India’s Polio Eradication Program [88, 89], and Ethiopia’s Health Extension Programme [64] provide successful models for community participation (S1 Appendix).

## Discussion

Engaging communities at the appropriate level and at right time requires a shared understanding of what CE means and what it entails. It was evident from the finding of our review that despite the ability of stakeholders to roughly describe the idea of CE in PHC, there was not a shared understanding of content of the work involved within or across different levels of engagement [6, 20]. A lack of universally agreed definitions and/or a different understanding of CE in the PHC context was inherent in the differing views about the level of CE. We found that CE is used in various ways and service providers employed multiple approaches to facilitate participation. These approaches of CE (and the meanings thereof) can generally be categorised into two: *CE as an end or a means to an end*. In CE, service providers employ a utilitarian-type participatory approach to gather community input to improve the delivery of established health programmes. In this approach, the parameters and power control are often in the hands of the health service. In contrast, CE as an end is dynamic and unpredictable, underpinned by a social justice-oriented participatory approach in which communities are empowered to participate, negotiate, influence control, and have a say in the decision-making process and outcome.

Regardless of the approaches of CE, the type of decision-making groups within which communities engage, their composition (i.e., representativeness in terms of number and socio-demographic diversity), and the extent to which they are allowed to define priorities and set the objectives are important factors that have a direct impact on the validity, usability, and outcome of CE initiatives in PHC setting [39]. Examples of common decision-making groups include advisory panels, governance boards, citizen councils, community forums, community health councils, patient and family advisory councils and boards, mixed advisory committees, patient organisations and other organisation committees, such as steering committees. Given that communities are not homogenous, adequate representation requires careful consideration of who is represented, and proactive identification of those who are not represented, which are often the least powerful members of the community. Modifying selection criteria without the involvement of the wider community affects representativeness of such community groups and impartiality of the process. Primary health workers can also manifest power by dominating the planning and budgeting processes. The marginalisation of some community groups and uneven power structures (with respect to educational status or gender) were identified as impacting the success of the CE initiative. Even though representation of women and marginalised people in community groups is ensured, it does not always mean that they have meaningful participation or will be given power in decision making [59]. In general, decentralisation of the decision-making process to the local level and ensuring that the community has an influence over the process (e.g., developing a sense of ownership, increased autonomy leadership) was found to improve participation in CE initiatives [37, 75]

The community values, needs and motivations provide a foundation to how CE initiatives take place, including how it is developed, delivered, and evaluated. Similar to the structural aspect of CE, the process or methods and mechanisms by which CE occurs can influence how well such CE activities impact health outcomes. This includes types and dynamics of the broad range of engagement approaches and activities, and presence of an on-going communication and two-way information sharing. The engagement approach describes the extent to which the community is allowed to provide leadership in decision-making, management, and planning of PHC services. Involvements, approaches and actions taken by organisational leaders to engage communities are key facilitators of successful CE. These include ‘top-down’ approach which involves institutional level commitment (often sponsored and led by healthcare system) to promote decision-making, and ‘bottom-up’ model–local champions-led initiatives which seeks to promote collaboration with providers to achieve a policy and/or practice change. Studies included in our study consistency reported that CE approaches based on ‘bottom-up’ model were more effective in improving participation and achieving the desired outcomes than ‘top-down’ model [45]. In contrast to less effective ‘top-down’ CE approach which maintains power within the hands of providers, empowerment-type participation of communities leads to an inevitable shift in power and control [16]. This can be perceived as a threat to entrenched power such as that assigned to health providers, local health facility managers and other stakeholders. Strategies to achieve active CE in PHC also need to recognise lay people’s difficulty navigating the health system and the often-disempowered nature of lay community’s relationships with health care providers. This power dynamics is manifested in several ways. For example, health providers may influence the composition of community groups such as health committees by modifying selection procedures to include educated persons.

The presence of an ongoing communication and two-way information sharing is also an important process of community engagement in PHC settings. Information flows within and across stakeholders through deliberative or non-deliberative processes [6]. Deliberation involves iterative discussions that enable participants to reflect, question, and provide points of view to uncover knowledge gaps and make consensus-based decisions. A two-way dialogue and communication strategy that is underpinned by transparency, respectfulness, reciprocity, inclusivity, and timely sharing of information was reported as essential characteristics of effective CE initiatives [21, 24, 25, 36, 40, 44]. It is also worth mentioning that the spectrum of responses from various stakeholders to engagement is partially dependent upon and is influenced by the extent to which communication is authentic and transparent, including how such information is used in informing decision-making.

Several studies highlighted the potential role of CE initiatives in improving decision making process and improving overall health outcomes by acting at various levels, including at an individual level (e.g., encouraging health behaviour change), at a family level (e.g., improved child vaccination), and at a societal level (e.g., developing social cohesion and building trust). Our review also identified several organisational, cultural, political, and contextual factors that affect the success of CE initiatives in PHC settings. In addition to consideration of socio-political context and power dynamics, successful implementation of CE requires organisational support. Structural issues, such as the lack of resources (financial and non-financial), facilitative leadership, training and capacity building have all been implicated in low level of community participation in CE initiatives. This calls for a paradigm shift in the cultural and organisational environment from one that treats CE as a voluntary exercise to one that mandates CE and a participatory approach in decision-making processes and service delivery. Such changes should include ensuring that service providers are well trained not only on medical issues, but also social aspects of care such as respect for and empowerment of patients and local communities. Our review has shown that only a small number of studies have examined the impact of CE initiatives on enhancing the utilization of maternal and child health services, such as antenatal and perinatal care [34, 48, 51]. This is a surprising finding, given the critical role of stronger, community-based PHC systems that foster community engagement and empowerment in ending preventable maternal and child deaths and achieving UHC [90, 91].

This is the first scoping review and realist synthesis to assess how and why community engagement initiatives implemented in PHC contexts result in improved decision-making processes and health outcomes. Although we have employed rigorous and standard approaches to describe and explain how, why and in what contexts CE initiatives work (or fail to work), our review is not without limitations. Given that most included studies were either scoping reviews, observational and/or qualitative studies, it was difficult to attribute any causal effects between CE interventions and associated outcomes. Much of the evidence relating to CE and health outcomes is from studies that document community-based participatory health interventions that involve community to achieve the aims of the intervention [34]. While participatory mechanisms are central to these health interventions, it is difficult to separate CE from the broader intervention process to evaluate its impact on health outcomes. In addition, the methods and outcome metrics used to evaluate the impact of CE in PHC y considerably depending on the approach taken in defining the scope and the overarching goal of the CE initiative. Most CE evaluation methods, which are often adapted and adopted from clinical medicine, focused mainly on the health impacts of CE, although the utility of these approaches is widely debated. Several studies highlighted the challenge of measuring the impact of CE, particularly in ‘bottom-up’ engagement where communities are empowered, and power control is relinquished. Of particular challenge is the difficulty in accounting for CE’s multi-faceted health and social dimensions, and in drawing causal linkages that explain how CE leads to a desired health outcome [24]. Conceptualising CE as a bounded, standardised ‘intervention’ can lead to framing the outcome measures only in terms of short-term attributes, while missing the bigger picture and multilevel effectiveness of CE. It is worth noting that only a few of the studies engaged communities in identifying and selecting appropriate outcomes and defining success for CE, including in studies where communities are given a role in shaping the development of the intervention programme itself [24, 38–40]. In addition, the majority of the studies lacked (or loosely defined) the purpose of the evaluation about the information needs of various stakeholders. Despite its limitations, findings from this review can inform donors, policymakers and implementers to design more effective community engagement initiatives to strengthen PHC and achieve UHC.

## Conclusions

Our review highlighted the potential role of CE initiatives in improving the decision-making process and overall health outcomes, and identified several organisational, cultural, political, and contextual factors that affect the success of CE initiatives in PHC settings. Awareness of and responding to the contextual factors will increase the chances of successful CE initiatives. There is also a need for methodological frameworks and CE evaluation methods to understand better, classify and evaluate associative mechanisms of community engagement implemented in the PHC setting and associated outcomes.

## Supporting information

S1 Appendix Supporting information (key words employed in the search strategy, characteristics of included studies, case studies and PRISMA-ScR checklist). (DOCX)
