# Supplementary material for: Community engagement initiatives in primary health care to achieve universal health coverage: A realist synthesis of scoping review
Source: PLoS One. 2023 May 3;18(5):e0285222. doi: 10.1371/journal.pone.0285222 (PMC10156058; doi:10.1371/journal.pone.0285222)
Supplement: S1 Appendix — (DOCX) [file pone.0285222.s001.docx]

S1 Appendix **.** Key words employed in the search strategy

| **Community engagement** | "**User** involvement" OR "User participation" OR "User contribution" OR "Community collaboration" OR "**Community** participation" OR "Community representative" OR "Community engagement" OR "Community input" OR "Community led" OR "Community involvement" OR "Lay representative" OR "Lay perspective" OR "**Lay** perception" OR "Lay involvement" OR "Lay participation" OR "Consumer participation" OR "consumer involvement" OR "consumer groups" OR "consumer driven" OR "consumer engagement" OR "patient perspective" OR "patient involvement" OR "patient participation" OR "**patient** representative" OR "patient engagement" OR "citizen participation" OR "**citizen** involvement" OR "citizen engagement" OR "citizen deliberation" |
| --- | --- |
| **UHC** | "Universal Health Care" OR "Health Equity" OR "Health Services Accessibility" OR "Quality of Health Care" OR "health coverage" OR "care coverage" OR "service coverage" OR "treatment coverage" OR "universal coverage" OR "universal health coverage" OR "UHC" OR "financing coverage" OR "Financial risk protection" OR "Financial hardship " OR "Financial protection" OR "Financial protection in health" OR "Efficiency" OR "Equity" OR "Responsiveness" OR "coverage" OR "effectiveness" OR "performance" |
| **PHC** | "primary health care" OR "intersectoral coordination" OR "multisectoral action*" OR multisectoralism OR "appropriate care" OR "comprehensive health care" OR "Equity" OR "Integrated care" OR "continuity of care" OR resilience |

**S2 Appendix**. Characteristics of included studies, n=67

| **Study** | **Country** | **Study type** | **Aim** | **Type of engagement** | **Main findings and author’s conclusions** |
| --- | --- | --- | --- | --- | --- |
| Alhassan et al 2015 (1) | Ghana | Randomized control trial | To examine the effect of community engagement interventions on patient safety and risk reduction efforts in 64 facilities | A systematic community engagement composed of i) recruitment and training of facilitators, and identification of existing community groups, ii) assessment of healthcare quality, iii) regional level validation and feedback sessions, iv) following up, and, v) rewarding best performing PHC facilities | - Systematic Community Engagement interventions significantly enhanced leadership processes and accountability. - The interventions did not seem to have significant effect on healthcare quality assessment outcomes, - Community groups that were gender balanced, religious/faith-based, and had structured leadership appeared to be better options for effective SCE in healthcare quality assessment. - Community engagement in healthcare quality assessment is a feasible client-centred quality improvement option. |
| Alhassan et al 2019 (2) | Ghana | Randomized control trial | To evaluate the impact of a community engagement intervention on maternal and child health services utilization in Ghana. | Systematic community engagement | - Intervention health facilities recorded significant improvements over control facilities in terms of average spontaneous vaginal deliveries per month per health facility, child immunizations and female condoms distribution. - Community engagement in health has the potential of improving utilization of maternal and child health services. There is the need for multi-stakeholder dialogues on complementing existing quality improvement interventions with community engagement strategies. |
| Alhassan et al 2016 (3) | Ghana | Randomized control trial | To assess the Impact of Community Engagement Interventions on health worker motivation and experiences with clients in primary health facilities in Ghana: | A structured use of existing community groups and associations to assess healthcare quality in health facilities. | - Community engagement in healthcare quality assessment could enhance client-provider relationships and potentially improve intrinsic motivation (non-financial) levels of staff. - Community-based approach to health worker motivation is a potential complementary strategy that needs policy deliberation to explore its prospects. - Albeit financial incentives remain critical sources of staff motivation, innovative non-financial approaches like SCE should complement the latter. |
| Bath et al 2013 (4) | Multiple | Literature review | To evaluate evidence of the impact of community participation in primary health care on health outcomes | Multiple | - The findings reveal a small but substantial body of evidence that community participation is associated with improved health outcomes. - There is a limited body of evidence that community participation is associated with intermediate outcomes such as service access, utilisation, quality and responsiveness. - Structural participation in the Aboriginal community-controlled sector has been shown to have an association with improved health outcomes in some instances. - PHC organisations and service providers are encouraged to consider participatory mechanisms where participation is an engaged and developmental process and people are actively involved in determining priorities and implementing solutions. |
| Bauhoff et al 2017 (5) | Tajikistan | Cognitive interviews | To assess consumers' understanding, interpretation of and preferences for displaying information for a health care report card in rural Tajikistan | Citizen report cards for PHC | - Findings are promising regarding the citizens' comprehension of health care report cards in rural Tajikistan, while underscoring the challenges of effectively providing health care performance information to communities. |
| Cheng et al 2014 (6) | Afghanistan | Experiences, insights and expert opinions | To demonstrate how the Afghan pre-migration experiences of PHC can affect engagement with Australian PHC services. | Multiple / unspecified | - Despite existing work to address these issues across the health sector, Afghans continue to face considerable challenges in utilising primary health care services. - Improved Afghan community engagement (e.g., community input, participatory community assessment, mobilising community resources and systems, comprehensive community communication strategies, and adaptability to the changing needs of the community) is an important strategy to address this gap. |
| Lunsford et al 2015 (7) | Ethiopia and Tanzania | Case studies | To provide case studies from Ethiopia and Tanzania regarding supporting close-to-community providers through a community health system approach | The Community Health System Strengthening (CHSS) model – an improvement approach which draws on existing formal and informal networks within a community to support close-to-community (CTC) providers and address gaps in community-based health services. | Ethiopia   - By participating on the community team, representatives became familiar with and comfortable referring people to health extension workers (compared to pre-implementation). During implementation, more pregnant women registered for ANC and tested for HIV; health extension workers conducted more postnatal visits; and more households had functioning latrines and proper latrine use increased.   Tanzania   - As a result of the model, increases in HIV testing and a reduction in patients lost to follow-up were observed.   CHSS model brings together existing networks within communities to support and lend legitimacy to CTC providers. This approach may result in sustainable community-based programmes |
| Okoli et al 2015 (8) | Nigeria | Cross-sectional survey | To assess the potential in the use of telecommunication technology as an effective way to engage members of the community in commodity stock monitoring in PHC. | Engaging Communities in Commodity Stock Monitoring Using Telecommunication Technology in PHC | - A sharp decline in the collection of user fees was observed, and there was a 10% rise in overall access to free health care services by beneficiaries. - This study reveals the effectiveness of mobile phones and indicates that telecommunication technologies can play an important role in engaging communities to monitor PHC stock levels as well as reduce the incidence of user fees collection |
| Reeve et al 2015 (9) | Australia | Case study, mixed methods | To describe the reorientation of a remote primary health-care service, in the Kimberley region of Australia, its impact on access to services and the factors instrumental in bringing about change. | A community-initiated health service partnership was developed between a community-controlled Aboriginal health organisation, a government hospital and a population health unit | - Evidence that community participation in health-care reform can lead to innovative primary health-care service delivery. - A community-led partnership providing sustainable health services resulted in increased access to primary health care. |
| Jones et al 2019 (10) | Australia | Descriptive study | To describe the design and implementation of a community engaged PHC strategy in rural Australia, the Primary Healthcare Registered Nurse: Schools-Based strategy | Community engaged PHC strategy in rural Australia, the Primary Healthcare Registered Nurse: Schools-Based strategy | - The strategy is underpinned by a cross-sector collaboration between a local health district, school education and a university department of rural health. - Although in the early stages of implementation, key learnings have been acquired and strategic, relationship, resource and workforce gains achieved. |
| Iyanda et al 2017 (11) | Nigeria | Cross-sectional study | To assess the process indicators and other factors influencing community participation in the delivery of primary health care. | Unspecified | - Community participation could take several forms; via mobilization, planning, needs assessment, advocacy, resource mobilization. - The overall community participation is wide as it situates clearly towards the empowerment end of the participation continuum. However, it further reveals that needs assessment and resource mobilization as indicators of participation needs to be improved upon to further widen the participation level. - Factors enhancing community participation in the delivery of primary health care include adequate female representation, incentives for community members for roles played in advocacy and mobilization, community involvement in planning and implementation, bottom-up approach to service delivery and collaboration with existing community structures. - Top-down approach to primary health care by some service providers impedes community participation. |
| Perry et al 2017 (12) | Multiple | Assessments of projects, programs, and research studies | Examine the common delivery strategies of community–based primary health care (CBPHC) projects that have demonstrated effectiveness in improving child mortality | Multiple; unspecified | Community collaboration was one of the six categories of strategies for program implementation were identified, all of which required working in partnership with communities and health systems. This includes   - Collaboration with or formation of village health committees and/or collaboration with local leaders - Formation and/or support of women’s groups - Sharing locally obtained health–related data with the community - Participatory Rural Appraisal (PRA) - Formation and/or support of microcredit programs for women - Involvement of older family members (men and grandparents/mothers–in–law) |
| Lodenstein et al 2017 (13) | Benin, Guinea and the DRC | Qualitative study | To explore the role of health facility committees in social accountability in primary health care | Health facility committees (HFCs) | - Most HFCs facilitate social accountability by engaging with health providers in person or through meetings to discuss service failures, leading to changes in the quality of services. - Social accountability practices are however often individualised and not systematic, and their success depends on HFC leadership and synergy with other community structures. - Most HFCs in this study offer a social accountability forum, but the informal and non-systematic character and limited community consultation leave opportunities for the exclusion of voices of marginalised groups. |
| Gurung et al 2018 (14) | Nepal | Qualitative study | To describe community representation in Nepal’s Health Facility Operation and Management Committees (HFMCs) and the degree of influence of community representatives in the HFMC decision-making processes | Health Facility Operation and Management Committees (HFMCs) | - The HFMC member selection process and decision-making within the committees were influenced by powerful elites. - Despite representation of the community on HFMCs, the depth of participation seems low. - There is also a need for orientation of service providers, including clinic managers, about the importance of community voice to strengthen the health system so that they are ready to share power and accommodate community concerns. |
| Kroneman et al 2018 (15) | Netherlands | Quantitative survey | To explore to what extent members of the community are willing to participate in the way their primary care practice is organized | Unspecified | - Half of the participants were ready to give their opinion on primary care and one-third reported willingness to participate in decision making. - Participants with previous experience in volunteering appeared more willing to spend time on participation. - This study showed that half of the respondents are willing to participate, but they are less sure about their ability to do so and that finding time to participate is seen as problematic. |
| Tierney et al 2018 (16) | Ireland | Focus groups and interviews | To analyse the implementation of community participation via primary care teams (PCTs) in Ireland. | Primary care teams (PCTs) | - Motivations to get involved in community participation work varied but were strong overall. - The implementation and sustainability of community participation on PCTs in Ireland will be limited unless (i) the functioning of PCTs is strong, (ii) there is increased confidence and clarity on community representatives’ roles among all health-care professionals, and (iii) more sophisticated methods for formal appraisal are used. |
| Weger et al 2018 (17) | Multiple | Review | To examine the barriers and enablers for engaging communities in the planning, designing, governing, and/or delivering of health and care services on the macro or meso level | Multiple; unspecified | - The review identified 8 guiding principles: Ensure staff provide supportive and facilitative leadership to citizens based on transparency, Foster a safe and trusting environment enabling citizens to provide input, Ensure citizens’ early involvement, Share decision-making and governance control with citizens, Acknowledge and address citizens’ experiences of power imbalances between citizens and professionals, Invest in citizens who feel they lack the skills and confidence to engage, Create quick and tangible wins, and Take into account both citizens’ and organisations’ motivations. - Any ‘meaningful participation’ of citizens can only be achieved if organisational processes are adapted to ensure that they are inclusive, accessible and supportive of citizens. |
| Folay et al 2019 (18) | USA | Mixed methods | To apply a Human Centred Design (HCD) approach to co-designing a comprehensive women’s health screening tool with community partners. | Human Centred Design (HCD) approach to co-designing screening tool | - Throughout the HCD sessions, community women, providers and clinic staff highlighted the impact of what they termed “Triple T: time, trust and talk” on the effectiveness of women’s health screening. - The amount of time providers have to spend with women during regular office visits is a structural constraint that a screening process may try to accommodate but cannot change. - Another important dimension to how screening may reinforce stereotypes that negatively impact health occurs when the screening process is deemed to create a brick wall from the patient’s perspective, and there is a lack of time to deal with the woman’s reason for the visit. |
| Makaula et al 2019 (19) | Malawi | Intervention study | To assess how best to use the community directed intervention (CDI) approach to strengthen locally identified PHC services at district level. | Community directed intervention (CDI) | - A CDI intervention in which the community discussed the health problems and possible intervention components from their own perspective, and took responsibility for implementation of intervention (i.e., designed the approach to implementing the intervention, identified the required resources from within their respective communities, planned how, when, where and by whom to implement the interventions, supervised and decided on what support to be provided to CDI implementers and how to monitor the processes, and reviewed the implementation process) resulted in improved accessibility of certain vital services at community level. |
| McEvoy et al 2019 (20) | Ireland | Qualitative study | To explore the levers and barriers to the implementation of community participation in primary healthcare as a routine way of working. | Unspecified | - Stakeholders did not always have a clear, shared understanding of the aims, objectives and benefits of community participation. Drivers/champions, and strong working partnerships, were considered integral to its initiation and implementation. - Participants emphasised the benefits of funding, organisational support, training and networking to enact relevant activities. - Health-promoting activities and healthcare consultation/information events were generally successful, but community representation on interdisciplinary Primary Care Teams proved more challenging. - Overall, participants were broadly positive about the impacts of community participation but were concerned about the scope to sustain the work without the ‘protected’ space and resources that the national Initiative afforded. |
| Rowe et al 2019 (21) | New Zealand | Qualitative study | To capture specific examples of community engagement by general practices, and to understand the barriers that prevent engagement | Multiple; unspecified | - The key findings from this study included the limited view of what is meant by a general practice community, the different perceptions of what is thought the community wants from general practice and valuable examples of ways some practices have engaged with their community. However, a common theme was that community engagement has a cost, especially a time cost, and that this is a major barrier to wider adoption. - General practices in the study do not think in terms of communities, and they do not have a systematic framework for engagement. Although local champions have generated some great initiatives, most practices seemed to lack a conceptual framework for engagement: who to engage with, how to engage with them, and how to evaluate the results of the engagement. |
| Cyril et al 2015 (22) | Multiple | Review | To examine the magnitude of the impact of CE on health and health inequalities among disadvantaged populations | Multiple; unspecified | - Key CE components that affected health outcomes included real power-sharing, collaborative partnerships, bidirectional learning, incorporating the voice and agency of beneficiary communities in research protocol, and using bicultural health workers for intervention delivery. - The findings suggest that CE models can lead to improved health and health behaviours among disadvantaged populations if designed properly and implemented through effective community consultation and participation. |
| Wilson et al 2019 (23) | UK and South Africa | Case studies | To examine programmes of work in public and community participation and involvement in UK and South Africa and share experiences. | South African community engagement project to reduce teenage pregnancy and HIV infection, and a national evaluation in England of public involvement in applied health research. | - In South Africa, community participation is seen as a route to decolonisation. It is also integral to the core functions of South African Higher Education Institutes, alongside teaching and research. - In the UK, there has also been a history of participation and involvement as part of a social rights movement, but notably public involvement has become embedded in publicly funded health research as a policy imperative - Partnership working in LMICs such as South Africa provides exemplars of innovative participatory approaches firmly shaped within the social justice paradigm, but with utilitarian outcomes for community |
| Blandón-Lotero et al 2020 (24) | Colombia | Mixed methods | To explain the conditions through which ethnic-rural territories of the Colombian Pacific coast participate in health to contribute to the generation of policies and programs in territories with similar conditions. | Multiple; unspecified | - Key findings include how the trajectory of social mobilization and existence of a robust community social fabric became two critical conditions for community participation in the context of social exclusion. - The presence of variables such as the implementation of PHC, guarantee of social rights, and trust in institutions, is underestimated as sufficient causal conditions for obtaining this result. - It is essential to recognize the existence, validity, and importance of processes, experiences, and resourcefulness of political natures, which aim at transforming the daily reality of the inhabitants of these communities. - These also set a potential space and scenario for managing the communities’ main problems, including health, in the absence of institutionality that guarantees access to their social rights. |
| Falisse et al 2020 (25) | Burundi | Pilot RCT | To examine the association between community-elected health facility committees and health facility performance indicators | Community-elected health facility committees (HFC) | - Training HFCs on their roles and providing them with information about the performances of their health facility (HF) do not lead to visible improvements in terms of social accountability, HF management, and use of and access to HF services. - Moreover, the information component ─or rather the one-off sharing of information with the HFC and failure to make such sharing more frequent─ may in fact exacerbate tensions at the HF. - The HFCs of Burundi appear sidelined, with little understanding of what their agents, the HF staff, are doing. |
| Haricharan et al 2020 (26) | South Africa | Mixed methods | To analyse whether the Western Cape Health Facility Boards and Committees Act is likely to result in effective and meaningful participation consistent with a PHC. | Health committees | - Current legislation is unlikely to lead to effective and meaningful participation. - First, the roles prescribed in the Act are narrowly defined. They resemble roles practised and are inconsistent with right-based and PHC frameworks. - Second, though the Act provides support, which the empirical research demonstrates is necessary, the support is insufficient, and often contingent. - Third, the Act conceptualizes health committees as structures appointed by the Provincial Minister of Health; a formation process likely to lead to structures that do not adequately represent community interests. |
| Haricharan et al 2021 (27) | South Africa | Mixed methods | To analyse health committees’ roles, their degree of influence in decision-making and factors impacting their participation. | Health committees | - The existing health committees faced sustainability and functionality challenges and primarily practised a form of limited participation. - Their decision-making influence was curtailed, and they mainly functioned as a voluntary workforce assisting clinics with health promotion talks and day-to-day operational tasks. - Several factors impacted health committee participation, including lack of clarity on health committees’ roles, health committee members’ skills, attitudes of facility managers and ward councillors, limited resources and support and lack of recognition. - To create meaningful participation, health committee roles should be defined in accordance with a PHC and human rights framework. - Their primary role should be to function as health governance structures at facility level, but they should also have access to influence policy development. - Consideration should be given to their potential involvement in addressing social determinants of health. Effective participation requires an enabling environment, including support, financial resources and training |
| Kweku et al 2020 (28) | Ghana | Quantitative study | To examine community involvement in and perceptions of Community-Based Health Planning and Services (CHPS) capacity to improve health outcomes of communities | Unspecified | - The level of community involvement in CHPS services is low. Ghana may not be able to attain the UHC goal by 2030 through CHPS implementation unless its level of community involvement is markedly improved. - Ghana’s health sector stakeholders should implement community engagement mechanisms that foster improved worker outreach, expanded use of community gatherings, and more active participation of traditional leaders and grassroots political representatives. |
| Questa et al 2020 (29) | LMICs | Umbrella review | To identify approaches to CE in communicable disease control, effectiveness of these approaches, mechanisms and factors influencing success. | Multiple; unspecified | - Approaches included: CE through peer education and community health workers, community empowerment interventions and more general community participation or mobilisation. - The authors found: i) significantly reduced neonatal mortality, HIV and other STIs, malaria incidence or prevalence, and in infant diarrhoea following community engagement interventions. - Factors influencing effectiveness of CE interventions included extent of population coverage, shared leadership and community control over outcomes. - Community engagement interventions may be effective in supporting CDC in LLMIC. Careful design of CE interventions appropriate to context, disease and community is vital. |
| Tiruneh et al 2020 (30) | Ethiopia | Mixed methods | To examine the effectiveness of participatory community solutions strategy on improving household and provider health care behaviors and practices: | Participatory community solutions strategy | - Interview participants said that the participatory design and implementation strategy helped them to realize gaps, identify real problems, and design appropriate solutions, and created a sense of ownership and shared responsibility for implementing interventions. - Community participation in planning and monitoring maternal and newborn health service delivery improves use of some high-impact maternal and newborn health services. |
| Alimehr et al 2021 (31) | Iran | Qualitative study | To identify challenges ahead of volunteers’ participation toward primary health‑care (PHC) system and providing managerial solutions | Volunteers’ participation toward PHC | - The most important challenges were categorized into six temporal, management, education, quality of services, motivation, and infrastructural themes. - Allocating special funds for the health volunteer program, paying attention to the problems of the health workers, providing motivational programs, holding regular retraining courses and providing educational software for health volunteers, holding regular training courses for health trainers and volunteers, and supporting the health volunteer program are among the solutions. |
| Ankomah et al 2021 (32) | Multiple | Scoping review | To identify the barriers and facilitators of Patient-Public Engagement for health system improvement in Sub-Saharan Africa: | Patient-Public Engagement | Individual and community level facilitators,   - Intrinsic motivation or people’s willingness to participate, - the opportunity PPE offers people to grow their personal and professional competencies as well as also gaining recognition and respect from their community - people’s sense of ownership for community‐based health programmes - aligning PPE to suit the cultural norms of the people as well as designing the programme to fit the local needs of the community, - the use of pre‐existing community structures such as the indigenous/traditional health care systems (e.g herbal healers, spiritualists and faith healers)   Individual and community level barriers   - lack of appropriate training, education and skill - Inadequate information or lack of clarity on the roles and responsibilities of community representatives - lack of logistical and financial support for the work of Community Health Volunteers - a lack of trust or support for community representatives |
| Haldane et al 2021 (33) | Multiple | Systematic review | To examine the evidence on outcomes of community participation in high and upper-middle income countries. | Multiple; unspecified | - Successful community outcomes were most evident among interventions that included outreach activities such as: health camps, community fairs, and partnerships with schools and religious groups; targeted interventions that delivered tailored and specific health knowledge; and interventions that encouraged relationship building with the wider community, - Failing to account for contextual learning can result in failure to work together to achieve goals, and this is especially important in vulnerable populations and those communities with a history of colonization and forced assimilation |
| Luisi et al 2021 (34) | Italy | Document analysis | To examine the Community participation and empowerment in PHC Emilia-Romagna | Multiple; unspecified | - Documents point to the need for democratic practice and shared decision-making power; third sector organisations are seen as salient community representatives and mediators. - However, the policies show only a vague conceptualisation of how to empower communities; moreover, strategies to promote participation of vulnerable groups are lacking. - Policies that consider the ambiguous role of the third sector, specify community empowerment, identify strategies to facilitate it and collaborate with vulnerable groups could be beneficial for further progress. |
| Oyeyemi et al 2021 (35) | Nigeria | Cross-sectional study | To ascertain the relationship between citizens’ participation and primary healthcare policy implementation challenges in Nigeria. | Citizens’ Participation | - Some of the challenges to PE are: Illiteracy, Access to social amenities, Financial responsibility, Insufficient manpower for engagement, Time factor for involvement, Lack of government engagement, and Poor overall leadership - The study recommends an integrated decision-making process that is bottom-up, which would support a sustainable healthcare system with enlightened citizens’ input towards the actualization of an effective PHC system. |
| Pedersen et al 2021 (36) | Multiple | Scoping review | To identify, chart and summarise public involvement methods in the planning, development and implementation of community health services, | Multiple; unspecified | - Two categories of public involvement methods were identified: the multiple methods approach and the single method approach. - the majority of the included studies used either participatory or community-based methodological approaches, which involved a variety of facilitation techniques. - The analysis identified five types of aims of public involvement with participatory or community-based approaches: (a) empowerment, (b) facilitation of critical dialogue between public citizens and policymakers, (c) identifying and understanding participants’ needs, (d) ranking and negotiation of the needs, and (e) participation in development, design, implementation and evaluation to ensure that interventions are feasible and acceptable. |
| SitieneiI et al 2021 (37) | Kenya | Multiple case study methodology | To examine  the implementation of community participation, through collaborative governance in primary health care facilities in Uasin Gishu County, Western Kenya | Unspecified | - Health facility committee meetings were most frequent when there were project funds, with discussions focusing mainly on construction projects as opposed to the day-to-day functioning of the facility. - Committee members with the strongest influence and power had political connections or were retired government workers. - There were no formal mechanisms for stakeholder forums and how these worked were unclear. - Drug stock outs, funding delays and unclear operational guidelines affected collaborative governance performance. |
| Belaid et al 2022 (38) | South Sudan | Document analysis | To examine how well the  Boma Health Initiative (BHI) policy addressed community participation in its policy formulation | Unspecified | - The BHI planners included inputs from communities without details on how the needs assessment was conducted at the community level, what needs were considered, and from which community. - The intended role of communities was to implement the policy under local leadership. - There was no information on how the Initiative might strengthen or expand local women’s leadership. - Official documents did not contemplate local power relations or address gender imbalance. - The policy approached households as consumers of health services. - Although the BHI advocated community participation to generate awareness, increase acceptability, access to services and ownership, the policy document did not include community participation during policy cycle |
| Hove et al 2022 (39) | South Africa | Narrative review | To provide evidence on forms, extents, contexts and dynamics of community participation in primary health care (PHC) and water governance in South Africa | Unspecified | - Health committees are not functioning effectively due to lack of clarity on roles, autonomy, power, support, and capacity. - Participation is not effective due to manipulation of spaces by elites, lack of capacity of previously disadvantaged individuals, inadequate incentives, and low commitment to the process by stakeholders. - Power and authority in decision-making, resources and accountability are key for effective community participation of marginalized people. |
| Karuga et al 2022 (40) | Sub Saharan Africa | Qualitative synthesis / review | To establish an in-depth understanding of how community members participate in primary health care through health committees in sub-Saharan Africa | Health committees | - Health committees contribute to community participation through holding primary health workers accountable, voicing their communities’ concern and mobilizing resources for health activities and projects. - Health committees lacked legitimacy because selection procedures were often not transparent and participatory. - Committee members were left out in planning and budgeting processes by health workers, who perceived them as insufficiently educated and trained to take part in planning. - Most health committees were male dominated, thus limiting participation by women. - Decision makers, health managers and advocates need to fundamentally rethink how health committees are selected, empowered and supported to implement their roles and responsibilities. |
| Sarrami-Foroushani et al 2014 (41) | Multiple | Meta review | To identify various dimensions of consumer and community engagement (CC)E-related strategies and offer a practical model to assist policymakers, practitioners and researchers. | Consumer and community engagement in | - Strategies for CCE vary in terms of their aim and type of proposed activity, as do the methods and tools which have been developed to support them. - Methods and tools include shared decision making, use of decision aids, consumer representation, application of electronic and internet-based facilities, and peer support. - The success of CCE is dependent on both the approach taken and contextual factors, including structural facilitators such as governmental support, as well as barriers such as costs, organisational culture and population-specific limitations. |
| Agegnehu et al 2022 (42) | Ethiopia | Cross-sectional study | To assess the level of knowledge regarding health rights and community participation in a town in Mizan-Aman, Ethiopia. | Unspecified | - The majority of the respondents in this study had low levels of community participation in primary healthcare. - A community‑based health education programme is needed to raise public awareness of heath rights and encourage community participation in primary healthcare activities. - Collaborating with academic and legal institutions may also help to facilitate community involvement in healthcare services. |
| CDI study group (43) | Multiple | Mixed-methods | To determine the extent to which the community-directed approach used in onchocerciasis control in Africa could effectively and efficiently provide integrated delivery of other health interventions. | Community-directed intervention (CDI) | - Selection of community implementers by communities is critical. - Community implementers are committed to serving their communities. - Community implementers are generally motivated by intrinsic incentives. - Community implementers expressed a desire for extrinsic financial incentives; however, the lack of financial incentives has not significantly affected their willingness to serve. - Participatory, consensus-building approaches to community mobilization are critically important. - Communities value implementers residing in the community. - Information and training increased awareness and ability to participate. - Community selection of implementers is important to enhance ownership and continuity. Where not selected by the community, lack of commitment emerged over time. - Continued participatory approaches remain necessary. |
| Kenny et al 2013 (44) | Multiple | Scoping review | To identify examples, in the international literature, of higher-level community participation in rural healthcare. | Unspecified | - Delegation of power to the community is challenging for some individuals or groups and power may be shared conditionally and withdrawn in times of conflict, - Reported outcomes included awareness of the health services provided and improved self-efficacy, social capital and accountability. - Benefits to community members included learning new skills, particularly in strategic planning, meeting facilitation, grant submission and leadership. - In an environment characterised by increasing interest in community participation in healthcare, greater understanding of the purpose, process and outcomes is a priority for research, policy and practice. |
| Lamb et al 2015 (45) | UK | Mixed methods | To develop and evaluate a model for community engagement component of the complex intervention | Multiple | - Community development approaches have many benefits, but perceptions of open-ended investment are a barrier. - The time-limited nature of a CE intervention provides an impetus to ‘do it now’, allowing stakeholders to negotiate their investment over time and accommodating their wider commitments. - Both tangible outcomes and recognition of process benefits were vital in maintaining involvement. - CE interventions can play a key role in improving accessibility and acceptability by engaging patients, the public and practitioners in research and in the local service ecology. |
| Makuala et al 2012 (46) | Malawi | Qualitative interview | To examine health service providers’ and beneficiaries’ perceptions on existing PHC practices, and their perspectives on official priorities and strategies to strengthen PHC. | Community-directed interventions approach | - Health service providers and consumers shared perceptions on the importance of intensifying community participation to strengthen PHC, particularly within the areas of provision of insecticide treated bed nets, home case management for malaria, management of diarrhoeal diseases, treatment of schistosomiasis and provision of food supplements against malnutrition. - Our study indicates that intensified community participation based on the CDI approach can be considered as a realistic means to increase accessibility of certain vital interventions at community level. |
| McEnvoy et al 2012 (47) | Ireland | Mixed-methods | To provide an overview of community participation in primary care, establishing the policy context in which a recent ‘Joint Initiative on Community Participation in Primary Health Care’ was developed in Ireland | Joint Initiative on Community Participation in PHC | The initiative resulted in:   - A notable shift in the views of many key individuals in the primary care sector about the place of community involvement in the planning of primary health care at a local level. - Joint approaches and working. - Improved capacity to identify community health needs and a shared understanding of the wider context of health. - Improved knowledge of PCT and community-led services, with several projects improving the availability of patient information sources within the local community through the development of a Directory of Services and the ongoing delivery of information workshops. - Economic benefits. |
| McGuire et al 2012 (48) | South Africa | Mixed methods | To examine the Entabeni Project and draw actionable recommendations | Entabeni Project as a CE intervention among CHWs | The Project provides concrete examples of how projects supporting CHWs can be successful, particularly in achieving ‘target- oriented’ objectives.   - Active involvement of community members in project design, inception and running - Training for CHWs was both medically and socially oriented - External change agents (ECAs) are crucial for mobilising support in marginalised communities, - Programmes wanting to achieve both ‘target- oriented’ and ‘empowerment- oriented’ outcomes need to be long term   Challenges identified included   - Cost - Lack of training among public sector employees |
| Neuwelt 2012 (49) | New Zealand | Qualitative study | To present key findings from a national study undertaken in the wake of the 2001 primary care reforms on the purpose and process of involving communities in primary health care. | Unspecified | - Views on community participation varied among different stakeholder groups in the sector. - Most described it as a complex process of relationship-building over time, and one that is quite distinct from consumer feedback processes in general practice. - For community representatives, it was a process of trust-building and information-sharing between communities and health professionals. - These relationships enabled people to feel comfortable seeking care, and professionals to mould services to people’s needs. - As citizens, members of disadvantaged communities are partners with general practices and PHOs, working with them to improve health equity by ensuring that services are responsive to their needs. |
| O’Mara-Eves et al 2015 (50) | Multiple | Meta analysis | To examine the effectiveness of community engagement in public health interventions for disadvantaged groups: | Unspecified | - There is solid evidence that community engagement interventions have a positive impact on a range of health outcomes across various conditions. - The results indicate that interventions employing incentives or skill development strategies tend to have higher effect size estimates than other strategies, while interventions with education approaches tend to be the least effective. - There is insufficient evidence to determine whether one particular model of community engagement is more effective than any other. |
| Waweru et al 2013 (51) | Kenya | Qualitative | To assess weather Health Facility Management Committees (HFMC) in Kenya are ready to implement financial management tasks | Health Facility Management Committees | - HFMC members and in-charges generally reported positive relationships, and HFMC members expressed high levels of motivation and job satisfaction. - Challenges included users’ low awareness of HFMCs, lack of training and clarity in roles among HFMCs, and some indications of strained relations with in-charges. - Such challenges are likely to be common to many similar settings and are therefore important considerations for any health facility-based initiatives involving HFMCs. |
| Goodman et al 2011 (52) | Kenya | Qualitative | To explore the nature and depth of managerial engagement of health facility committees at the facility level in two rural districts in this Coastal setting, and how this has contributed to community accountability | Health Facility Management Committees | - HFCs were generally functioning well and played an important role in facility operations. The breadth and depth of engagement had reportedly increased after the introduction of direct funding of health facilities which allowed HFCs to manage their own budgets. - Although relations with facility staff were generally good, some mistrust was expressed between HFC members and health workers, and between HFC members and the broader community, partially reflecting a lack of clarity in HFC roles. - Moreover, over half of exit interviewees were not aware of the HFC's existence. Women and less well-educated respondents were particularly unlikely to know about the HFC. |
| Singh 2021 (53) | India | Systematic review | To examine the evidence on community engagement and communication from studies that report on program outcomes in India | Unspecified | - This review adds to this evidence base supporting the utility of community participation in yielding positive outcomes at the organizational, community, and individual level across a wide range of health domains. - Our findings present process and community outcomes as necessary to achieving robust health outcomes. This supports the notion that CE do not happen as a linear progression, but rather consists of complex processes influenced by an array of contextual factors. - Highlights the need for more robust program evaluations of community engagement initiatives that measure long-term outcomes and cost-effectiveness, in more settings globally. |
| South et al 2014 (54) | NA | Viewpoint | To stimulate debate on the evaluation of community engagement where it is a major component of public health programmes. | Unspecified | - Community engagement is too often conceptualised for evaluation purposes as a bounded, standardised intervention ‘done to’ communities and the effects of independent social action by communities are difficult to capture. - There is a need for a paradigm shift from viewing the involvement of communities as an errant form of public health action, to seeing communities as an essential part of the public health system. |
| Akinyemi OO et al 2021 (55) | Nigeria | cross-sectional survey | To assess factors that enhanced or impeded the implementation of community engagement strategies using the Nigerian polio programme as a point of reference | Unspecified | - Almost half of the participants identified the process of conducting the PEI program and social environment as the most important internal and external contributor to implementing community engagement activities in the community, respectively. - The economic environment was the most frequently reported challenge among the external challenges to implementing community engagement activities. - Community engagement strategies were largely affected by the factors relating to the process of conducting the polio programme, the economic environment and the social context. |
| Hammanyero et al 2018 (56) | Nigeria | Mixed methods | To evaluate the outcome of CE activities, to examine the methods and processes that helped to increase OPV and penta3 immunization coverage in areas of implementation | Unspecified | - The systematic implementation of a CE strategy that focused on planning and working with community structures and community engagers in immunization activities assisted in increasing OPV and penta3 immunization coverage |
| Gilmore t al 2020 (57) | Multiple | systematic literature review | To identify how community engagement is used for infectious disease prevention and control during epidemics. | Unspecified | - Six main community engagement actors were identified: local leaders, community and faith-based organisations, community groups, health facility committees, individuals and key stakeholders. - These worked on different functions: designing and planning, community entry and trust building, social and behaviour change communication, risk communication, surveillance and tracing, and logistics and administration. - Well-implemented community engagement strategies can be used to support designing of interventions, building trust and community entry, social and behaviour chance communication, risk communication, surveillance and contract tracing, and logistical and administrative support during COVID-19 prevention and control responses. |
| Adhikari et al 2016 (58) | Multiple | systematic literature review | To examine the population coverage and community engagement in programmes of mass anti-malarial drug administration | Unspecified | - Community engagement activities included providing health education and incentives, using community structures (e.g. existing hierarchies or health infrastructure), mobilizing human resources, and collaborating with government at some level (e.g. ministries of health). - Community engagement was often a process involving various activities throughout the duration of the intervention. - Further research is needed to understand the factors that influence population coverage and adherence in mass anti-malarial administrations and the role community engagement activities and approaches play in satisfactory participation |
| Brunton et al 2017 (59) | Multiple | Scoping review | To develop a conceptual framework which informs understanding about what makes an effective (or ineffective) community engagement intervention. | Unspecified | - This identified multiple dimensions by which community engagement interventions may differ. - Diverse combinations of intervention purpose, theory and implementation were noted, including: ways of defining communities and health needs; initial motivations for community engagement; types of participation; conditions and actions necessary for engagement; and potential issues influencing impact. - Some dimensions consistently co-occurred, leading to three overarching models of effective engagement which either: utilised peer-led delivery; employed varying degrees of collaboration between communities and health services; or built on empowerment philosophies. |
| Snow et al 2018 (60) | Multiple | Scoping review | To develop a heuristic model to assist planners to engage patients who are not traditionally included in healthcare planning. | Unspecified | - The review identified power and gender as barriers to participation, and generated suggestions to support diverse populations both to attend patient engagement events and to participate meaningfully. - Engaging marginalized populations cannot be reduced to a single defined process, but instead needs to be understood as an iterative process of fitting engagement methods to a particular situation. Underlying this process are principles for meaningfully engaging marginalized people in healthcare planning. |
| Durey et al 2016 (61) | Australia | Mixed methods | To evaluate a unique strategy of community engagement between local Aboriginal people and health providers across five districts in Perth, Western Australia. | Unspecified | - The engagement was driven and owned by the Aboriginal community, captured a broad range of views and increased Aboriginal community participation in decisions about their healthcare. - It built community capacity through regular community forums and established DAHAGs comprising local Aboriginal community members and health service representatives who met quarterly and were supported by the Aboriginal Health Team at the local Population Health Unit. - Participants reported health services improved in community and hospital settings, leading to increased access and trust in local health services. |
| Dawson et al 2004 (62) | Australia | Mixed methods | To provide an overview of the key policy and strategic direction documents shaping consumer and community participation in PCPs, and critically analyses these documents against the ladder of participation | Unspecified | - The initial in-principle commitment and practical support given by the state government to PCPs to develop and implement consumer participation represented a commitment to consumer participation at the higher levels of the ladder of participation. - The funding and reporting guidelines for PCPs—based on outcomes/impact measures—locate consumer participation at the lower levels of the ladder of participation. |
| Preston et al 2010 (63) | Multiple | Review | To disentangle the conceptual gaps in community engagement, and clarify our common understanding of community participation | Unspecified | - Although there is some evidence of benefit of community participation in terms of health outcomes, the authors found only a few studies demonstrating higher levels of evidence. However, it is clear that absence of evidence of effect is not necessarily the same as absence of an effect. - The paper focus on areas of debate and lack of clarity in the literature. Improving our understanding of community participation and its role in rural primary health care service design and delivery will increase the likelihood of genuine community–health sector partnerships and more responsive health services for rural communities. |

**S3 Appendix.** Selected case studies of successful CE approaches to promote participation in PHC

The table below summarised selected case studies of successful CE approaches to promote participation in PHC. The selected case studies represent diverse countries (high income vs low-income countries) and patient groups (e.g., CE in marginalised peoples – Aboriginal community-controlled health services), and various engagement mechanisms.

| **Characteristics** | **Examples of community engagement approaches and/or community-led health programs** | | | |
| --- | --- | --- | --- | --- |
|  | **Ethiopia’s Health Extension Programme (HEP)**  (51) | **Australia’s Aboriginal Community Controlled Health Services (ACCHS) Model of Care** (59, 77) | **Ireland’s Joint initiative on Community Participation in PHC**  (43, 76) | **India's Polio Eradication Program (78, 79)** |
| **Context** | HEP is one of the most innovative community-based health programs in Ethiopia and assumes that access to and quality of PHC in rural communities can be improved through transfer of health knowledge and skills to households. | ACCHS model of care aims to close the gap i.e., the disparity in health between Aboriginal and Torres Strait Islander people in Australia and their non-Indigenous peers, thereby improving service delivery to Aboriginal population in rural and remote areas. | Ireland developed a Joint Initiative to support the involvement of disadvantaged communities and groups in the development of PHC services at local level, with the aim of informing national policy and practice. Nineteen projects were funded under the joint initiative. | In 2003, the USAID–funded CORE Group Polio Project placed community mobilization coordinators at the frontline of polio eradication effort in Uttar Pradesh, the most populous Indian state and one of the first to become overtly hostile toward the polio program. |
| **Objective of CE** | - To improve access to and quality of PHC in rural communities through transfer of health knowledge and skills to households. | - To overcome the challenges of delivering PHC to a dispersed, highly disadvantaged Aboriginal population in a very remote area. | - To support and test models of community participation in primary care with a view to informing national policy and practice. | - To improve low vaccination rates associated with communities’ lack of trust in a polio eradication campaign and in the government health system. |
| **Main actors, and composition of CE groups** | - Government of Ethiopia; volunteer women’s development army; public sector, community, and development partners. | - Community-controlled Aboriginal health organisation, government hospitals and a population health unit. - Powerful coalition was created including community leaders and elders. | - Community Development Projects, Community and Voluntary Forums, Local Development Companies, a Family Resource Centre and a Local Regeneration Agency. | - CORE Group Polio Project (a consortium of NGOs with national technical input) and community leaders. |
| **Underpinning principles** | - The community is not only a service user but also a service provider. - Community behaviour is changed step by step: training early adopters first, then moving to the next group that is ready to change (i.e., diffusion model). | - Power is explicitly vested in local communities. - Aboriginal people must be included in leadership positions and decision making processes. - It’s important for clients to feel safe, welcome, and empowered. - An Aboriginal workforce delivering PHC ensures a culturally safe environment - Priority of PHC services determined by communities. | - The participation of communities and groups who experience poverty and social exclusion is essential to the development of PHC services. - Community participation is a process that takes time and requires resources, particularly in building mutual learning and respect, promoting dialogue and creating realistic goals, expectations and actions. - Multi-sectoral approaches are essential if community participation in primary care is to achieve long-term outcomes. | - Programs, no matter how vital, cannot be pushed into communities without proper information preceding them. - Community engagement needs to be on the agenda of any public health program from the start and not viewed as a separate objective. |
| **Contents of CE (type, and dynamics extent, time, timing)** | - Volunteers work with their neighbours to teach and provide a role model for basic health and sanitation behaviours - Model families further influenced their communities, by piloting innovative activities. - Trained ‘model’ families influenced their neighbours via pre-existing organisations already formed for mutual practical support (e.g., *idir* and *mahber).* | - Sense of urgency occurred naturally due to high mortality and morbidity. - Dedicated funding for PHC enabled a more appropriate staffing mix and additional primary care services to be provided through increased workforce capacity. - Committed local leadership overcome bureaucratic barriers to new models of care - Structural changes enabled by the delineation of roles and responsibilities through the partnership agreement. | Activities include:   - Joint project planning through a Community Participation Steering Group. - Projects benefited from having a key person(s) to oversee and manage the project locally. - Carried out a mapping of local community and resources in the project area. - Information sharing activities were carried out. - Processes of capacity building were put in place (and carried out training) to build engagement, knowledge and the confidence to participate. - Engaged in some form of community consultation through public meetings, workshops or focus groups. - A renewed focus has been given to the broader determinants of health, and - Projects put in place a strategy to sustain community participation | - Community mobilizers spent time with women, explaining the advantages of immunization, dispelling mistruths, and discussing other health and sanitation issues. - Communities shared their concerns and collaborated with community leaders to identify solutions. - Children were used as mobilizers, carrying positive vaccination messages through communities. - Famous Bollywood stars contributed to a very concerted and focused mass media program |
| **CE outcome** | - “Model household” status achieved by many throughout the country. - Created greater awareness of how to prevent communicable diseases - Changed community’s attitudes and behavioural practices in preventive aspects of maternal and child health. - Created synergy among public sector, community, and development partners | - Community consultation resulted in trusting relationships and clarity around a shared vision with local PHC providers. - Reduced barriers to access to health care – coverage of the Aboriginal population higher than 60% outside major metropolitan centres. - Improved individual health outcomes for Aboriginal people. | - Local community involvement has enhanced the understanding of the broader determinants of health and has uncovered and identified new community health needs. - Improved capacity, motivation and commitment from the community to participate in primary care | - Increased participation in and understanding of polio eradication activities - Expanded health services and greater government responsiveness to community health needs |

**S4 Appendix**. Preferred Reporting Items for Systematic reviews and Meta-Analyses extension for Scoping Reviews (PRISMA-ScR) Checklist

| **SECTION** | **ITEM** | **PRISMA-ScR CHECKLIST ITEM** | **REPORTED ON PAGE #** |
| --- | --- | --- | --- |
| **TITLE** | | | |
| Title | 1 | Identify the report as a scoping review. | 1 |
| **ABSTRACT** | | | |
| Structured summary | 2 | Provide a structured summary that includes (as applicable): background, objectives, eligibility criteria, sources of evidence, charting methods, results, and conclusions that relate to the review questions and objectives. | 2 |
| **INTRODUCTION** | | | |
| Rationale | 3 | Describe the rationale for the review in the context of what is already known. Explain why the review questions/objectives lend themselves to a scoping review approach. | 3 |
| Objectives | 4 | Provide an explicit statement of the questions and objectives being addressed with reference to their key elements (e.g., population or participants, concepts, and context) or other relevant key elements used to conceptualize the review questions and/or objectives. | 4 |
| **METHODS** | | | |
| Protocol and registration | 5 | Indicate whether a review protocol exists; state if and where it can be accessed (e.g., a Web address); and if available, provide registration information, including the registration number. | 4 |
| Eligibility criteria | 6 | Specify characteristics of the sources of evidence used as eligibility criteria (e.g., years considered, language, and publication status), and provide a rationale. | 4 |
| Information sources* | 7 | Describe all information sources in the search (e.g., databases with dates of coverage and contact with authors to identify additional sources), as well as the date the most recent search was executed. | 4, 5 |
| Search | 8 | Present the full electronic search strategy for at least 1 database, including any limits used, such that it could be repeated. | 5 |
| Selection of sources of evidence† | 9 | State the process for selecting sources of evidence (i.e., screening and eligibility) included in the scoping review. | 5 |
| Data charting process‡ | 10 | Describe the methods of charting data from the included sources of evidence (e.g., calibrated forms or forms that have been tested by the team before their use, and whether data charting was done independently or in duplicate) and any processes for obtaining and confirming data from investigators. | 5 |
| Data items | 11 | List and define all variables for which data were sought and any assumptions and simplifications made. | 5 |
| Critical appraisal of individual sources of evidence§ | 12 | If done, provide a rationale for conducting a critical appraisal of included sources of evidence; describe the methods used and how this information was used in any data synthesis (if appropriate). | 5 |
| Synthesis of results | 13 | Describe the methods of handling and summarizing the data that were charted. | 5 |
| **RESULTS** | | | |
| Selection of sources of evidence | 14 | Give numbers of sources of evidence screened, assessed for eligibility, and included in the review, with reasons for exclusions at each stage, ideally using a flow diagram. | 5 |
| Characteristics of sources of evidence | 15 | For each source of evidence, present characteristics for which data were charted and provide the citations. | 6 |
| Critical appraisal within sources of evidence | 16 | If done, present data on critical appraisal of included sources of evidence (see item 12). | 6,7 |
| Results of individual sources of evidence | 17 | For each included source of evidence, present the relevant data that were charted that relate to the review questions and objectives. | 6, 7 |
| Synthesis of results | 18 | Summarize and/or present the charting results as they relate to the review questions and objectives. | 6 - 10 |
| **DISCUSSION** | | | |
| Summary of evidence | 19 | Summarize the main results (including an overview of concepts, themes, and types of evidence available), link to the review questions and objectives, and consider the relevance to key groups. | 11 |
| Limitations | 20 | Discuss the limitations of the scoping review process. | 12, 13 |
| Conclusions | 21 | Provide a general interpretation of the results with respect to the review questions and objectives, as well as potential implications and/or next steps. | 14 |
| **FUNDING** | | | |
| Funding | 22 | Describe sources of funding for the included sources of evidence, as well as sources of funding for the scoping review. Describe the role of the funders of the scoping review. | 14 |

JBI = Joanna Briggs Institute; PRISMA-ScR = Preferred Reporting Items for Systematic reviews and Meta-Analyses extension for Scoping Reviews.

* Where *sources of evidence* (see second footnote) are compiled from, such as bibliographic databases, social media platforms, and Web sites.

† A more inclusive/heterogeneous term used to account for the different types of evidence or data sources (e.g., quantitative and/or qualitative research, expert opinion, and policy documents) that may be eligible in a scoping review as opposed to only studies. This is not to be confused with *information sources* (see first footnote).

‡ The frameworks by Arksey and O’Malley (6) and Levac and colleagues (7) and the JBI guidance (4, 5) refer to the process of data extraction in a scoping review as data charting*.*

§ The process of systematically examining research evidence to assess its validity, results, and relevance before using it to inform a decision. This term is used for items 12 and 19 instead of "risk of bias" (which is more applicable to systematic reviews of interventions) to include and acknowledge the various sources of evidence that may be used in a scoping review (e.g., quantitative and/or qualitative research, expert opinion, and policy document).

*From:* Tricco AC, Lillie E, Zarin W, O'Brien KK, Colquhoun H, Levac D, et al. PRISMA Extension for Scoping Reviews (PRISMAScR): Checklist and Explanation. Ann Intern Med. 2018;169:467–473. [doi: 10.7326/M18-0850](http://annals.org/aim/fullarticle/2700389/prisma-extension-scoping-reviews-prisma-scr-checklist-explanation).
